# Aldosterone-Mediated Atrial Remodeling and Atrial Fibrillation: Molecular Mechanisms, Translational Insights and Implications for Cardiac Surgery

**DOI:** 10.3390/ijms27219466

**Published:** 2026-10-23

**Authors:** Debora Emanuela Torre, Carmelo Pirri

**Affiliations:** 1 Department of Cardiac Anesthesia and Intensive Care Unit, Cardiac Surgery, Ospedale dell’Angelo, Mestre, 30174 Venice, Italy; deboraemanuela.torre@aulss3.veneto.it; 2 Department of Neurosciences, Institute of Human Anatomy, University of Padova, 35121 Padova, Italy

**Keywords:** aldosterone, mineralcorticoid receptor, atrial fibrillation, atrial remodeling, primary aldosteronism, postoperative atrial fibrillation, cardiac surgery

## Abstract

Atrial fibrillation (AF) has heterogeneous mechanisms, including structural atrial cardiomyopathy, electrical remodeling and primary ion-channel abnormalities. Aldosterone–mineralcorticoid receptor (MR) signaling may contribute to an arrhythmogenic atrial substrate through redox-sensitive, inflammatory, profibrotic and electrophysiological pathways; however, their direct demonstration in human atria varies. Primary aldosteronism is associated with excess AF risk, but circulating aldosterone, renin-independent secretion, tissue MR activation and MR-antagonist exposure are not interchangeable biological measures. This narrative review critically distinguishes evidence from human atrial tissue, atrial experimental systems and extrapolation from other cardiovascular models, with particular emphasis on the cardiac surgical setting. Preoperative aldosterone has been associated with postoperative atrial fibrillation (POAF), whereas perioperative MR-antagonist studies have reported discordant findings and observational analyses remain susceptible to confounding. We propose a testable, rather than established, two-hit framework in which a pre-existing endocrine-associated atrial substrate may interact with perioperative inflammation and hemodynamic stress. The review identifies gaps in endocrine phenotyping and the need for adequately powered prospective and interventional studies before aldosterone-directed POAF prevention can be recommended.

## 1. Introduction

Atrial fibrillation (AF) is a common arrhythmia arising from heterogeneous electrical triggers and substrates. Structural atrial cardiomyopathy, including fibrosis, inflammation and altered cell-to-cell conduction, can facilitate AF, but inherited ion-channel abnormalities may also contribute to AF independently of overt structural remodeling [1,2,3].

Aldosterone–mineralcorticoid receptor (MR) signaling has emerged as one potential contributor to atrial injury through oxidative, inflammatory, profibrotic and ion-channel pathways [4,5,6]. Clinical observations in primary aldosteronism (PA) further suggest arrhythmic risk beyond that attributable to elevated blood pressure alone [7,8]. Cardiac surgery provides a clinically distinct setting: a pre-existing vulnerable substrate is exposed to cardiopulmonary bypass, ischemia–reperfusion, inflammation, altered atrial loading and autonomic activation, all recognized contributors to postoperative atrial fibrillation (POAF) [9,10,11,12]. While previous reviews have extensively addressed the molecular and clinical relationship between aldosterone/MR signaling and atrial fibrillation, the present review specifically examines their potential implications in cardiac surgery. Its distinctive contribution is the critical integration of experimental and human atrial evidence with perioperative clinical studies, highlighting discrepancies between randomized and observational findings, distinguishing different aldosterone-related endocrine phenotypes and proposing a testable “two-hit” framework for postoperative atrial vulnerability.

## 2. Evidence Linking Aldosterone/MR Signaling to Atrial Remodeling

### 2.1. Aldosterone Signaling: Molecular Mechanisms Driving Atrial Remodeling

#### 2.1.1. Mineralcorticoid Receptor Signaling

Growing evidence indicates that the arrhythmogenic effects of aldosterone cannot be explained solely by its clinical role in sodium and water homeostasis [4]. Instead, aldosterone acts as a pleiotropic signaling molecule capable of modulating multiple cellular processes involved in cardiovascular remodeling. These effects are primarily mediated through activation of the mineralcorticoid receptor (MR), a ligand-activated transcription factor belonging to the nuclear receptor superfamily that is widely expressed not only in epithelial tissues but also in cardiomyocytes, cardiac fibroblasts, vascular endothelial cells, vascular smooth muscle cells and adipose tissue. The broad distribution of the MR provides the biological basis for the extensive cardiovascular actions of aldosterone beyond renal electrolyte regulation [13]. Under physiological conditions, aldosterone diffuses across the plasma membrane and binds to cytoplasmic MR, which is maintained in an inactive conformation through association with heat shock proteins and other molecular chaperones. Ligand binding induces a conformational change that promotes receptor dissociation from the chaperone complex, dimerization and subsequent nuclear translocation. Once inside the nucleus, the activated MR interacts with specific hormone response elements within targeted genes, recruiting transcriptional co-activators and chromatin remodeling complexes that regulate the expression of numerous proteins involved in inflammation, oxidative stress, extracellular matrix turnover, cellular proliferation, apoptosis and tissue fibrosis. These genomic effects typically develop over several hours and contribute to the long-term structural remodeling observed during chronic aldosterone excess [14,15]. In addition to these classical genomic actions, aldosterone also exerts rapid non-genomic effects that occur within minutes and are independent of de novo gene transcription or other protein synthesis. These responses may be mediated by the classical MR through interactions with membrane-associated signaling complexes, while MR-independent mechanisms have also been described. Rapid aldosterone signaling involves several intracellular pathways, including c-Src/EGFR-dependent activation of ERK1/2 and other MAPKs, PI3K/Akt signaling and protein kinase C, with downstream effects on intracellular Ca^2+^ homeostasis, nitric oxide signaling, ion transport and cellular redox balance. These rapid pathways can interact with classical genomic MR signaling and with other neurohormonal systems, particularly angiotensin II signaling, thereby potentially amplifying cardiovascular remodeling [16,17,18,19,20]. Aldosterone and angiotensin II exert synergistic rather than independent effects on cardiovascular remodeling, with reciprocal amplification of oxidative stress, inflammatory signaling and profibrotic pathways. Angiotensin II enhances aldosterone synthesis through stimulation of the adrenal zona glomerulosa while simultaneously increasing MR expression and downstream signaling within cardiovascular tissues [20,21,22]. Conversely, aldosterone potentiates angiotensin II-mediated vascular injury by promoting nicotinamide adenine dinucleotide phosphate (NADPH) oxidase activation, reactive oxygen species (ROS) generation, endothelial dysfunction and reduced nitric oxide bioavailability. This bidirectional interaction establishes a self-perpetuating feed-forward loop that accelerates tissue remodeling independently of systemic blood pressure [23,24,25,26,27]. Emerging evidence further indicates that MR activation may occur even in the absence of markedly elevated circulating aldosterone concentrations. Oxidative stress, hyperglycemia, obesity, chronic kidney disease and inflammatory mediators can induce ligand-independent MR activation through post-translational receptor modifications and altered intracellular signaling. In parallel, local synthesis of aldosterone within cardiovascular tissues has been proposed, although the quantitative contribution of extra-adrenal aldosterone production in humans remains incompletely defined. Nevertheless, these observations support the concept that inappropriate MR activation may develop across a broad spectrum of cardiovascular diseases, extending well beyond classical primary aldosteronism [28,29,30,31,32,33]. An important distinction should therefore be maintained between circulating aldosterone concentrations, autonomous or renin-independent aldosterone secretion and tissue-level MR activation. These phenomena are biologically interconnected but not interchangeable. Circulating aldosterone provides an endocrine biomarker, whereas MR activation reflects the downstream biological response and may be influenced by ligand-independent mechanisms, the local tissue environment and receptor sensitivity [13,28,29,30,31,32].

#### 2.1.2. Cellular Mechanisms of Atrial Remodeling

Aldosterone-associated mineralcorticoid receptor (MR) signaling can increase oxidative stress through several experimentally characterized steps, but evidence must be attributed to its biological system. In vascular cells, Callera et al. demonstrated aldosterone-induced c-Src-dependent activation of p38 mitogen-activated protein kinase and NADPH oxidase [34]. In aldosterone-exposed rat myocardium, Sun et al. linked oxidative stress to myocardial inflammation [35]. In other cardiovascular models, rapid kinase signaling, NADPH oxidase activation and mitochondrial redox amplification provide complementary mechanisms [16,17,18,19,20,36,37]. NADPH oxidase transfers electrons from NADPH to oxygen to generate superoxide, which can be converted to hydrogen peroxide and influence redox-sensitive signaling. These experiments should not be presented as direct proof of an uninterrupted aldosterone-MR-NOX2 pathway in human atrial cardiomyocytes.

Experimental studies have implicated aldosterone in NLRP3-related inflammatory signaling in vascular [38] and renal models [39]. Independently, enhanced NLRP3 inflammasome signaling has been associated with atrial fibrillation in experimental atrial models, without establishing aldosterone as the initiating stimulus [40]. Profibrotic mechanisms, including TGF-β-related signaling, have also been investigated in experimental cardiac and atrial fibrosis models [41,42,43,44]. However, these findings do not demonstrate a unified aldosterone-dependent inflammatory and profibrotic pathway in human atrial tissue. The highest level of atrial anatomical relevance derives from studies using human atrial specimens; Lavall et al. characterized MR-associated fibrotic signaling and 11β-hydroxysteroid dehydrogenase type 2 expression in human atrial tissue [45], while Danesh et al. identified MR-responsive cell populations in donor human atria during experimental cold preservation and reperfusion [46]. These observations support atrial target engagement or association, not a causal link between a preoperative endocrine phenotype and clinical POAF. Reil et al. used chronic aldosterone exposure in experimental atrial models to link hormone exposure to fibrosis and conduction abnormalities [47]. By contrast, mechanisms involving specific NOX isoforms, inflammasome activation, mitochondrial bioenergetics and endothelial injury are frequently derived from ventricular, vascular, renal or aldosterone-specific AF systems [34,35,38,39,48,49,50]. Collagen deposition and extracellular matrix reorganization can contribute to conduction heterogeneity [51,52,53,54,55,56,57], but a causal inference should be restricted to the model and intervention actually studied.

#### 2.1.3. Electrical Remodeling

Electrical remodeling provides arrhythmic mechanisms that may occur with or without extensive fibrosis. In aldosterone-exposed atrial experimental models, changes in calcium currents and cellular electrophysiology have been reported [58,59,60]. Calcium overload and spontaneous diastolic calcium release can facilitate delayed afterdepolarizations and triggered activity in AF more generally [61,62,63], but these latter mechanisms have not all been established as aldosterone-specific effects in human atria. Redox-sensitive RyR2, SERCA2a and sodium-calcium exchange pathways also contribute to broader AF biology; direct aldosterone-specific validation in human atrial tissue remains incomplete [63,64,65]. Altered refractoriness, conduction and connexin distribution may interact with structural remodeling; this interaction must not be presented as universal across phenotypes [66,67,68,69,70,71]. Inherited or acquired primary electrical abnormalities may initiate AF independently of detectable structural atrial disease. Variants affecting cardiac sodium and potassium channels and other excitability-related proteins have been associated with familial or early onset AF [1,3]. Such channelopathies provide an important counterexample to any assertion that AF as a whole is the consequence of fibrosis or progressive atrial cardiomyopathy. Aldosterone-mediated remodeling is therefore discussed here as one potentially modifiable contributor to AF susceptibility, not as an obligatory final pathway.

Intercellular electrical coupling represents an additional component of the arrhythmogenic atrial substrate. Coordinated impulse propagation throughout the atria depends on the integrity and spatial organization of gap junctions, specialized intercellular channels predominantly composed of connexin 40 (Cx40) and connexin 43 (Cx43). These proteins enable rapid electrical communication between adjacent cardiomyocytes and contribute to synchronous atrial activation. MR-dependent remodeling may influence gap-junction organization and intercellular electrical coupling through structural, inflammatory and oxidative mechanisms; however, available data regarding aldosterone-specific changes in connexin expression and distribution remain limited and inconsistent. In atrial fibrillation more broadly, connexin remodeling appears to involve not only changes in protein abundance but also abnormal lateralization and heterogeneous spatial redistribution of gap junctions, particularly Cx40 and Cx43. These alterations, together with interstitial fibrosis that disrupts normal myocardial continuity, can increase conduction heterogeneity and anisotropy, promoting localized conduction slowing and block and thereby facilitating re-entrant arrhythmogenesis [47,67,68,69,70]. Structural remodeling and electrical remodeling therefore evolve as closely interconnected processes rather than as entirely independent events. Progressive collagen deposition disrupts normal cell-to-cell electrical continuity and increases conduction heterogeneity, while sustained rapid atrial activation during AF promotes calcium loading, ion-channel remodeling, oxidative stress and additional cellular dysfunction. These changes progressively reinforce one another, creating a self-perpetuating substrate in which electrical instability promotes further structural remodeling and structural abnormalities, in turn, facilitate arrhythmia maintenance. This reciprocal interaction provides the mechanistic basis for the progressive nature of atrial fibrillation and supports the well-established concept that “atrial fibrillation begets atrial fibrillation” [71]. In the setting of chronic aldosterone excess, persistent MR activation may further reinforce the reciprocal interaction between structural and electrical remodeling, favoring progression toward a more persistent arrhythmogenic atrial substrate.

**Figure 1:**
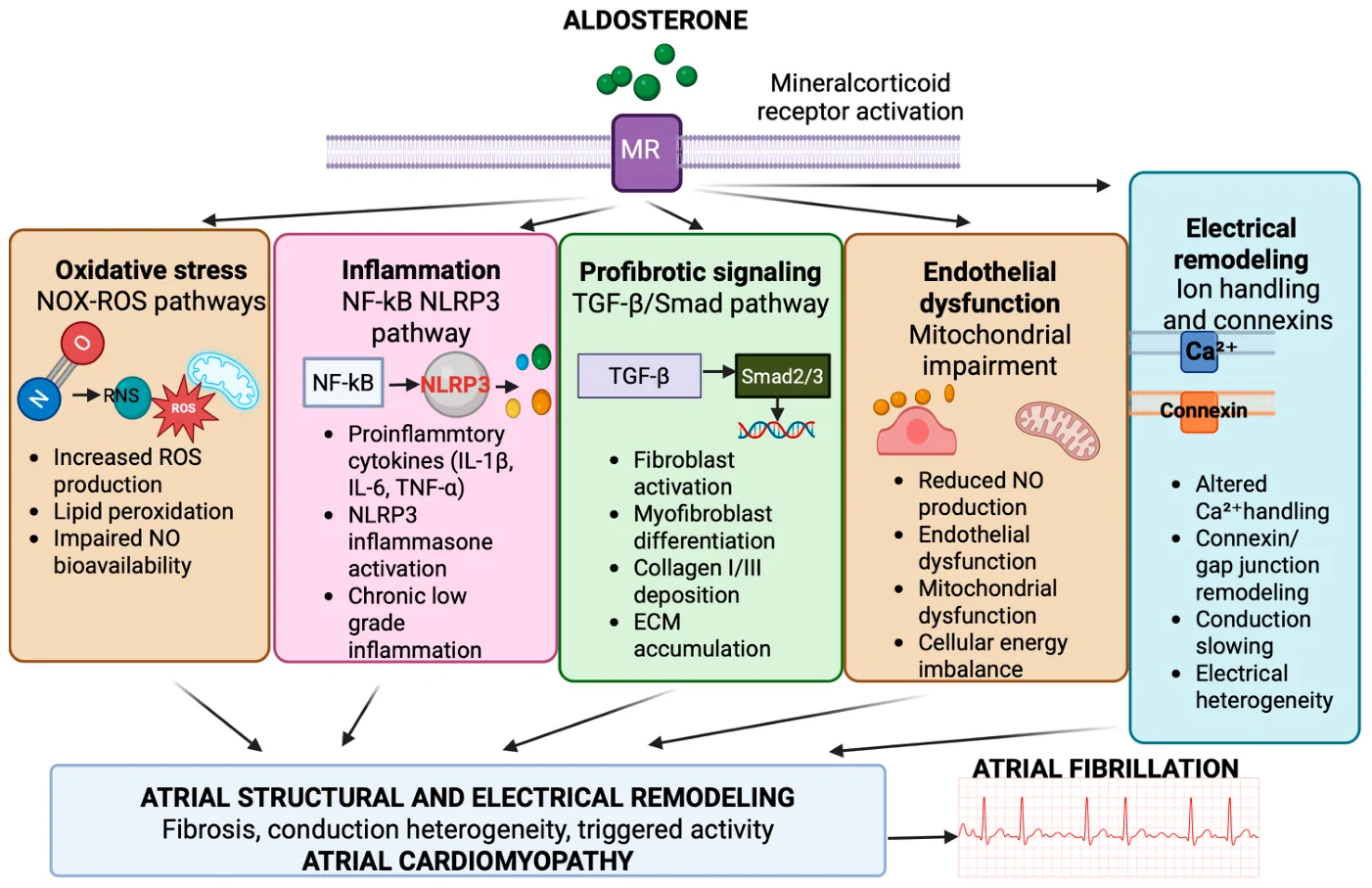
Molecular mechanism of aldosterone/MR signaling in atrial remodeling and atrial fibrillation. MR: mineralcorticoid receptor; ROS: reactive oxygen species; RNS: reactive nitrogen species; NOX: NADPH oxidase; NF-kB: nuclear factor kappa B; NLRP3: NOD-like receptor family pyrin domain-containing 3; TGF-β: transforming growth factor beta; ECM: extracellular matrix; Ca^2+^: calcium; IL: interleukin; Smad 2/3: SMAD family member 2/3 (intracellular mediators of TGF-β signaling). Created in BioRender. Pirri, C. (2026) https://BioRender.com/zxdc9lw (accessed on 9 September 2026).

Experimental fibroblast-cardiomyocyte cross-talk, connexin redistribution and inflammatory signaling provide additional potential substrates for conduction heterogeneity [67,68,69,70,71,72,73,74]. Nevertheless, direct electrical coupling between fibroblasts and atrial cardiomyocytes is much better characterized in vitro than in intact human atrial tissue. These mechanisms are thus retained as plausible adjuncts rather than established dominant causes of clinical AF (Figure 1).

### 2.2. From Experimental Models to Human Disease

#### 2.2.1. Experimental Evidence

**Figure 2:**
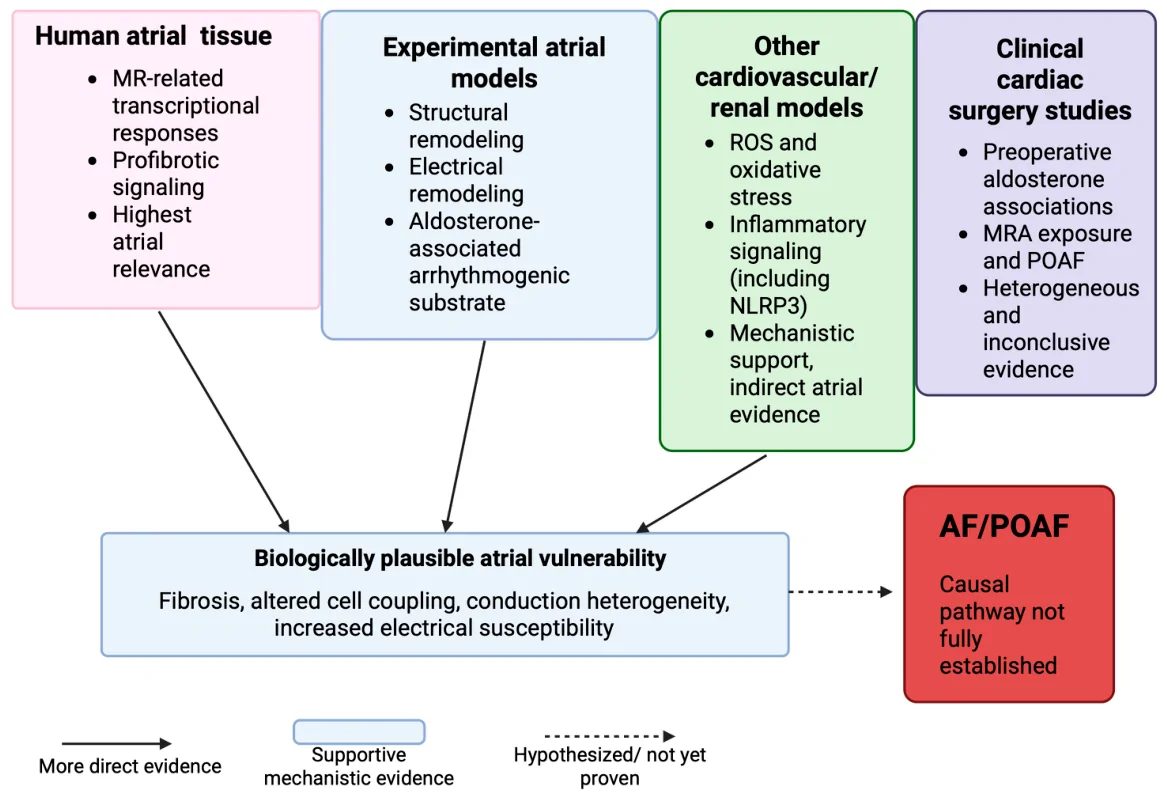
Evidence-graded links between aldosterone/MR signaling, atrial remodeling and atrial fibrillation. Created in BioRender. Pirri, C. (2026) https://BioRender.com/pheg47r (accessed on 9 September 2026).

Experimental evidence provides complementary but non-equivalent tiers of support (Figure 2). Chronic aldosterone exposure in atrial animal models has been associated with interstitial fibrosis, fibroblast accumulation, conduction disturbances and increased atrial arrhythmia susceptibility [47]. In human atrial tissue, Lavall et al. examined fibrosis-associated MR signaling and 11 β-HSD2 expression [45]. These atrial-specific observations should be distinguished from experimental studies demonstrating aldosterone-associated fibrosis and functional alterations in broader cardiovascular or animal atrial models [35,75,76,77]. Complementary genetic studies involving cell-specific MR deletion have demonstrated the contribution of cardiomyocyte and myeloid MR signaling to cardiovascular remodeling, although these findings cannot be directly extrapolated to human atrial arrhythmogenesis [78,79]. Pharmacological MR antagonism or genetic manipulation in experimental systems supports pathway involvement but neither establishes a clinical preventive effect of POAF.

The mechanistic evidence linking aldosterone/MR signaling to atrial remodeling derives from different experimental settings and should therefore be interpreted according to its biological context. Aldosterone-induced ROS generation through c-Src-dependent NADPH oxidase activation has been demonstrated in vascular cells [34], while aldosterone-associated oxidative stress and inflammatory responses have been reported in experimental rat myocardium [35]. Although these findings support a biologically plausible redox-mediated mechanism, they do not establish the complete signaling pathway in human atrial tissue. More directly relevant evidence comes from experimental atrial models demonstrating aldosterone-associated structural and electrophysiological remodeling [47] and from studies involving human atrial tissue or donor hearts documenting MR-related transcriptional and profibrotic responses [45,46]. Similarly, the involvement of NLRP3 inflammasome signaling has been demonstrated in renal and other cardiovascular experimental models [38,39], whereas studies of atrial fibrillation have identified inflammasome activation without necessarily establishing aldosterone as its upstream trigger [40]. Taken together, these findings support the biological plausibility of interconnected oxidative, inflammatory, profibrotic and electrophysiological mechanisms. However, the extent to which these pathways form a unified causal sequence in human atrial remodeling particularly in the development of POAF remains to be established.

#### 2.2.2. Clinical Evidence

Clinical evidence provides complementary support for the potential relationship between aldosterone excess and atrial fibrillation. Primary aldosteronism is common and frequently underrecognized among patients with hypertension, as documented by epidemiological studies and clinical guidelines [80,81,82,83]. More specifically, observational cohorts and pooled analyses have reported an increased risk of AF in patients with primary aldosteronism compared with those with essential hypertension [7,8,84,85]. These clinical findings are consistent with experimental evidence linking aldosterone/MR signaling to atrial remodeling. However, observational associations do not establish that the molecular mechanisms demonstrated in experimental models directly cause AF in humans. The main experimental, clinical and translational studies supporting this relationship, together with their principal findings and limitations, are summarized in Table 1.

#### 2.2.3. The Cardiovascular and Atrial Phenotype of Aldosterone Excess

A consistent observation across clinical studies is that patients with PA exhibit a greater burden of target-organ damage than patients with essential hypertension, even when comparisons are adjusted for age, sex and blood pressure levels [8]. Left ventricular hypertrophy, impaired diastolic function, increased myocardial mass, vascular stiffness and myocardial fibrosis have all been reported more frequently in the setting of inappropriate aldosterone secretion [90,91,92]. Although the magnitude of these differences varies according to patient selection, disease severity, diagnostic criteria and treatment status, the overall pattern strongly supports the existence of aldosterone-mediated cardiovascular injury that is not solely attributable to hypertension. The atria appear particularly vulnerable to this process. Chronic exposure to aldosterone excess is associated with structural changes that include left atrial enlargement, impaired atrial mechanical function and abnormalities in atrial strain reflecting progressive alterations in myocardial compliance and tissue architecture [93,94,95]. Left atrial enlargement represents only one component of a broader remodeling process. Human atrial studies have documented interstitial fibrosis and alterations in myocardial architecture in patients with atrial fibrillation [45,67]. In patients with primary aldosteronism, speckle-tracking echocardiography has demonstrated impaired atrial deformation and increased estimated atrial stiffness, suggesting that functional abnormalities contribute to the atrial phenotype [93]. Consequently, conventional echocardiographic measures of atrial size alone may not adequately characterize the underlying substrate, which encompasses structural, contractile and electrophysiological alterations [57]. In patients with aldosterone excess, these abnormalities may develop gradually and remain clinically silent until an additional trigger reveals the underlying vulnerability. The association between PA and atrial fibrillation therefore likely reflects the cumulative consequences of long-standing molecular and structural remodeling rather than an isolated proarrhythmic effect of elevated circulating aldosterone [57]. An important aspect of this phenotype is the apparent dissociation between blood pressure and cardiovascular injury. Patients with comparable office blood pressure values may exhibit substantially different degrees of myocardial remodeling depending on the presence and biological activity of aldosterone excess. This observation has contributed to the hypothesis that aldosterone should be considered an independent modifier of cardiovascular risk [96,97]. Chronic MR activation may amplify the effects of mechanical stress by promoting fibrosis, inflammation, oxidative injury and microvascular dysfunction, thereby lowering the threshold at which conventional cardiovascular risk factors translate into clinically relevant myocardial disease [7].

#### 2.2.4. Aldosterone Excess and the Risk of Atrial Fibrillation

Among the cardiovascular manifestations of PA, the association with AF is particularly compelling. Observational studies and meta-analyses have consistently reported a substantially higher prevalence and incidence of AF among patients with PA compared with primary hypertension, with a magnitude of risk that cannot be fully explained by differences in conventional cardiovascular risk factors or blood pressure alone. These clinical observations are consistent with the mechanistic framework described above, in which chronic aldosterone/MR activation promotes atrial fibrosis, chamber remodeling, impaired diastolic and atrial mechanical function, oxidative and inflammatory signaling and electrophysiological instability. Aldosterone-associated cardiovascular remodeling may occur in patients with other established risk factors for AF, including hypertension-related pressure overload, obesity, aging, diabetes and obstructive sleep apnea [5,8,80,96,98]. Although primary aldosteronism has been associated with increased risk of AF, current evidence does not establish whether aldosterone-related remodeling interacts synergistically with these additional risk factors or independently accelerates the progression from subclinical atrial abnormalities to clinically manifest arrhythmia [8].

The temporal relationship between aldosterone excess and AF is also clinically relevant because PA frequently remains unrecognized for prolonged periods, allowing cardiovascular and atrial remodeling to develop before endocrine evaluation is undertaken [99]. The occurrence of AF in patients with hypertension may therefore represent an additional clinical clue to inappropriate aldosterone secretion. Current guidelines increasingly emphasize broader screening for primary aldosteronism and specifically recommend reconsidering PA testing when otherwise unexplained AF develops after a previously negative evaluation [83]. Importantly, the relationship between aldosterone signaling and AF appears to extend beyond the traditional dichotomy between overt PA and primary hypertension. Growing evidence supports a continuum of renin-independent aldosterone secretion, ranging from subtle or subclinical autonomous aldosteronism to overt PA. Individuals at the milder end of this spectrum may not fulfill conventional diagnostic criteria for PA but may nevertheless exhibit inappropriate aldosterone secretion relative to renin status and potentially clinically relevant MR activation [100,101]. Recent prospective evidence further supports this concept, demonstrating that higher aldosterone-to-renin ratios across the population are independently associated with incident AF [102]. These observations challenge a strictly binary classification of aldosterone physiology and raise the possibility that clinically relevant aldosterone/MR-mediated cardiovascular risk may remain undetected in some individuals currently classified as having primary hypertension.

#### 2.2.5. Can Aldosterone-Mediated Atrial Remodeling Be Modified?

Evidence that MR blockade modifies an arrhythmogenic substrate varies by population and endpoint. In PA cohorts, adrenalectomy and medical MR antagonism have been associated with different subsequent AF risk; residual renin suppression during MRA treatment may identify incomplete physiological blockade, but treatment assignment is not randomized [7,84,103,104,105,106,107]. Randomized evidence outside cardiac surgery is mixed and should not be considered direct evidence for POAF prevention. In EMPHASIS-HF, among patients with mild systolic heart failure and no baseline atrial fibrillation/flutter, eplerenone reduced new-onset atrial fibrillation/flutter (25/911, 2.7% vs. 40/883, 4.5%; HR 0.58, 95% CI 0.35–0.96; *p* = 0.034) in a prespecified secondary analysis; however, a covariate-adjusted estimate was less precise and not statistically significant (HR 0.71, 95% CI 0.49–1.05; *p* = 0.087). Neither analysis establishes benefit in surgical patients [88]. By contrast, IMPRESS-AF randomized 250 individuals with permanent AF and preserved ejection fraction to spironolactone 25 mg/day or placebo for two years and found no significant improvement in peak oxygen consumption, E/e′ or quality of life; incident AF prevention was not its primary objective [89]. These trials investigate different diseases and endpoints and therefore neither directly contradict nor confirm an aldosterone-specific POAF mechanism. The role of MR antagonists in perioperative prevention requires surgery-specific evidence, accounting for renal function, hyperkalemia, treatment duration and hemodynamic indications.

#### 2.2.6. Beyond Overt Primary Aldosteronism: Subclinical and Unrecognized Aldosterone Excess

The term “occult aldosterone excess” is used here only as a conceptual umbrella for potentially unrecognized endocrine abnormalities, not as a diagnosis or a biomarker positive group. Three categories must remain distinct. First, undiagnosed primary aldosteronism denotes patients who meet established biochemical diagnostic criteria but have not yet been recognized clinically [83]. Second, milder renin-independent aldosterone secretion may display a continuum of suppressed renin and aldosterone that is inappropriate for the physiological context without satisfying conventional thresholds [99,100,101,102,108,109]. Third, increased tissue MR signaling may occur without demonstrably elevated circulating aldosterone and cannot be classified as aldosterone excess on the basis of receptor activity alone [13,28,29,30,31,32]. Single plasma aldosterone measurements, adrenal autonomy testing and preoperative MR-antagonist prescription interrogate different aspects of this biology; they are not interchangeable proxies. Medication exposure, salt intake, potassium, renal function and sampling conditions also affect aldosterone-renin interpretation [83]. Current evidence has not established the prevalence of these three categories or validated their incremental predictive value for POAF. Accordingly, any proposed interaction with surgical inflammation or ischemia–reperfusion remains a testable hypothesis, not a proven risk phenotype. Clinical studies conducted in patients undergoing cardiac surgery provide the most directly relevant evidence regarding the relationship between aldosterone/MR signaling and POAF. Observational studies have reported associations between preoperative aldosterone concentrations or MRA use and POAF, while randomized trials investigating perioperative MR antagonism have yielded conflicting results [12,46,86,87]. Complementary studies using human atrial tissue have provided insights into the molecular effects of MR signaling and its pharmacological inhibition, although these findings do not establish the clinical efficacy of MR antagonists in preventing POAF [45,46]. Evidence from the non-surgical AF population, experimental cardiac models and heart failure trials further supports the potential involvement of aldosterone/MR signaling in atrial arrhythmogenesis but its applicability to the perioperative setting remains uncertain [7,88,89].

### 2.3. Aldosterone-Mediated Atrial Remodeling in Cardiac Surgery

#### 2.3.1. Interaction Between Pre-Existing Atrial Substrate and Perioperative Injury

**Figure 3:**
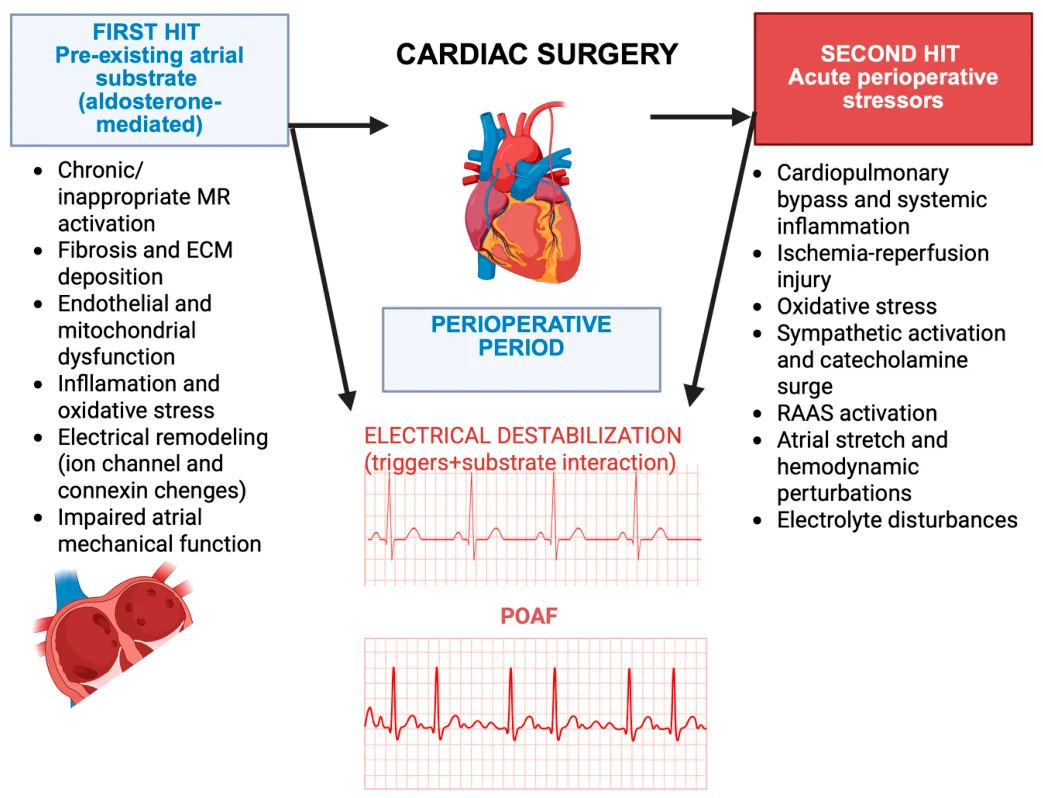
Two-hit model of aldosterone-mediated susceptibility to postoperative atrial fibrillation. MR: mineralcorticoid receptor; ECM: extracellular matrix; POAF: postoperative atrial fibrillation; RAAS: renin–angiotensin–aldosterone system. Created in BioRender. Pirri, C. (2026) https://BioRender.com/rqwwgku (accessed on 9 September 2026).

POAF is a multifactorial complication resulting from the interaction between pre-existing atrial vulnerability and acute perioperative stressors, including cardiopulmonary bypass, ischemia–reperfusion injury, systemic inflammation, autonomic imbalance and hemodynamic and electrolyte disturbances [9,10,11,110,111]. Within this framework, chronic aldosterone excess may contribute to the development of a vulnerable atrial substrate through structural and electrical remodeling. Cardiac surgery may subsequently provide additional triggers that facilitate the onset of AF. This interaction forms the basis of the proposed “two-hit” model (Figure 3). However, although biologically plausible, this hypothesis has not yet been directly demonstrated in cardiac surgical patients. Furthermore, perioperative changes in circulating RAAS hormones do not necessarily reflect MR activation within atrial tissue or establish a causal relationship with POAF [112,113].

#### 2.3.2. Clinical and Interventional Evidence in Cardiac Surgery

Cardiac surgical studies provide a crucial but heterogeneous body of clinical evidence of the proposed perioperative framework (Table 1).

In 137 elective coronary artery bypass grafting (CABG) patients with preserved ejection fraction, Alexandre et al. reported higher preoperative plasma aldosterone in patients subsequently developing POAF (183 versus 143 pmol/L; POAF incidence 24.8%) [12]. A single circulating measurement provides an association, not confirmation, of primary aldosteronism, renin-independent secretion or atrial MR activation. Potential confounding by operative characteristics, atrial disease and perioperative exposures limits causal inference. Pretorius et al. conducted a double-blind placebo-controlled randomized trial in 445 patients undergoing elective cardiac surgery. Treatment was started 4–7 days before surgery: spironolactone 25 mg/day, ramipril or placebo. POAF occurred in 25.9%, 27.8% and 27.2% of participants respectively (*p* = 0.95) [86]. The negative result limits simple assertion of effective short-course prophylaxis, although treatment duration, population heterogeneity and power to detect a modest benefit are relevant limitations. Farzaneh et al. reported a lower POAF incidence after spironolactone 50 mg/day for two weeks before CABG versus placebo (7.7% versus 20%; odds ratio 0.33; *p* = 0.042) in a single-blind trial of 130 patients [87]. The favorable finding is hypothesis-generating; the sample and number of events were limited, the masking was not double-blind and the result should be replicated in broader surgical populations with standardized rhythm monitoring. In the retrospective Mayo Clinic study by Danesh et al., 19042 cardiac surgery patients were evaluated; 1:3 propensity matching yielded 298 preoperative mineralcorticoid receptor antagonist (MRA) users and 894 non-users, with POAF occurring in 19.8% and 31.8% respectively [46]. An additional diuretic-treated comparison was reported, but propensity matching cannot eliminate confounding by indication, particularly differences in heart failure, renal function, concomitant therapy, atrial substrate, adherence and exposure duration. Complementary experiments conducted on human donor atrial tissue demonstrated that canrenone modulates MR-related transcriptional responses to ischemia–reperfusion stress, providing further evidence of the potential involvement of MR signaling in atrial injury [46]. However, these molecular findings do not establish that preoperative MRA treatment prevents POAF nor do they indicate that patients receiving MRAs necessarily have underlying aldosterone excess, since these agents may be prescribed for several cardiovascular indications independently of the patient’s endocrine phenotype. Overall, the available evidence remains inconclusive. The absence of a significant preventive effect in the double-blind randomized trial by Pretorius et al. contrasts with the favorable results reported by Farzaneh et al. and the observational association identified by Danesh et al. Differences in study design, patient selection, treatment duration, MRA dosage and potential confounding factors may partly explain these discrepancies. Although experimental findings support the biological plausibility of MR involvement in atrial remodeling and perioperative stress responses, current clinical evidence is insufficient to establish a causal preventive effect of MR antagonism on POAF or to recommend routine preoperative MRA initiation solely for this purpose. Further adequately powered randomized trials are needed to determine whether selected cardiac surgical patients, potentially identified through endocrine or atrial phenotyping may benefit from targeted antagonism.

#### 2.3.3. Occult Aldosterone Excess and Postoperative Atrial Fibrillation

The concept of “occult aldosterone excess” requires careful interpretation, as it may encompass different biological and clinical conditions. Some patients may have previously undiagnosed primary aldosteronism, while others may exhibit milder forms of renin-independent aldosterone secretion. Furthermore, increased mineralcorticoid receptor activity may occur even in the absence of documented aldosterone excess. Although these conditions may share common cardiovascular consequences, they should not be considered equivalent. Importantly, available studies in cardiac surgical patients have not systematically investigated the relationship between preoperative aldosterone and renin profiles, atrial MR activation and the subsequent development of POAF [12,46,86,87]. Therefore, the potential contribution of these different endocrine and molecular phenotypes to POAF risk remains uncertain, and “occult aldosterone excess” should currently be regarded as a conceptual rather than an established diagnostic or prognostic entity. Future prospective studies should assess preoperative aldosterone and renin concentrations under standardized conditions, accounting for medications and other factors that may influence their interpretation. These measurements should be integrated with established clinical and perioperative risk factors and systematic postoperative rhythm monitoring to determine whether aldosterone-related abnormalities independently contribute to POAF susceptibility. Importantly, preoperative treatment with MR antagonists should not, by itself, be considered evidence of underlying aldosterone excess or adequate MR blockade.

## 3. Discussion

The principal contribution of this review is to bring together endocrine, atrial and perioperative evidence to examine whether aldosterone-related vulnerability may influence POAF. These lines of evidence are complementary but not interchangeable. Clinical studies of primary aldosteronism consistently support an association between an established endocrine disorder and increased atrial fibrillation risk [7,8,84]. Such findings, however, do not establish that underdiagnosed primary aldosteronism or milder renin-independent aldosterone secretion independently predicts POAF in patients undergoing cardiac surgery. Likewise, a higher circulating aldosterone concentration, suppression of renin, tissue mineralcorticoid receptor (MR) activation and the prescription of an MR antagonist (MRA) represent different observations and cannot be used as substitutes for one another. The mechanistic literature strengthens biological plausibility while also defining its limits. Aldosterone-associated activation of c-Src and NADPH oxidase has been described in vascular cells [34] and oxidative stress-associated inflammation has been observed in experimental rat myocardium [35]. Direct experimental atrial work demonstrates that sustained aldosterone exposure can promote structural changes and conduction abnormalities [47]. Studies involving human atrial tissue further describe MR-related profibrotic pathways and cell-specific transcriptional responses [45,46]. Nevertheless, the available studies do not establish one continuous aldosterone-MR-ROS-inflammasome-fibrosis-POAF causal sequence in human surgical patients. For example, inflammasome findings derived from non-atrial aldosterone models [38,39] should be distinguished from atrial AF experiments in which aldosterone was not necessarily the initiating stimulus [40]. Moreover, AF can develop through mechanisms other than progressive structural remodeling, including inherited ion-channel abnormalities [3]. The clinical evidence specific to cardiac surgery remains the critical test of this proposed translation. Alexandre et al. identified an association between preoperative plasma aldosterone concentrations and subsequent POAF, but this did not establish an underlying endocrine diagnosis or atrial MR activation [12]. In interventional studies, a double-blind trial did not find a reduction in POAF after a short preoperative course of spironolactone, whereas a smaller single-blind trial reported a favorable effect after a longer course [86,87]. Danesh et al. found an association between preoperative MRA exposure and lower POAF incidence in a propensity-matched retrospective cohort, together with ex vivo human atrial responses to canrenone [46]. The molecular experiments provide evidence of a drug-responsive tissue pathway; these experiments neither remove confounding from the clinical comparison nor prove that MRA users had aldosterone excess. Differences in study design, dosing, duration, populations, outcome surveillance and residual confounding prevent these results from being combined into a definitive preventive claim. Likewise, MRA findings in heart failure or non-surgical AF populations cannot be treated as proof of perioperative benefit [88,89]. These observations provide the rationale for proposed “two-hit” framework. In this model, a pre-existing atrial substrate potentially influenced by chronic endocrine signaling constitutes the first component, while cardiopulmonary bypass, ischemia–reperfusion, inflammation, autonomic changes and perioperative loading or electrolyte disturbances represent additional triggers [9,10,11,110,111]. The model is intended to explain one possible subgroup of POAF susceptibility rather than all cases of postoperative arrhythmia. Perioperative changes in the renin–angiotensin–aldosterone system have been documented during cardiac surgery [112,113], but circulating hormone fluctuations alone do not demonstrate atrial MR activation or show that aldosterone mediates the subsequent arrhythmia. Direct evidence connecting a defined preoperative endocrine phenotype, atrial tissue response and rigorously ascertained POAF within the same patient population is currently lacking.

The expression “occult aldosterone excess” should therefore remain a hypothesis-generating description and not be presented as a validated diagnostic or prognostic entity. Previously unrecognized primary aldosteronism, milder renin-independent secretion and enhanced tissue MR signaling in the absence of documented aldosterone excess should be considered separately. Whether any of these features add predictive information beyond age, atrial disease, hypertension, comorbidity and operative factors is unknown. Routine aldosterone-renin testing solely to predict POAF or initiation of MRA therapy solely for POAF prophylaxis cannot currently be recommended on the basis of the studies discussed here. Assessment and treatment of established primary aldosteronism should continue to follow the relevant endocrine indications [83].

Finally, the interpretation of this synthesis is limited by its narrative design, the heterogeneity of the included populations and endpoints and the predominance of observational or non-atrial mechanistic evidence for several proposed pathways. No formal systematic risk-of-bias evaluation or quantitative pooling was undertaken. Some studies investigate incident or recurrent non-surgical AF, whereas others address POAF, molecular readouts or tissue preservation responses; the outcomes are not equivalent. The review therefore identifies a biologically and clinically relevant research question, but does not claim that the proposed endocrine-atrial phenotype, or a preventive effect of perioperative MR blockade, has been established.

## 4. Literature Search and Narrative Synthesis

This narrative review was conducted to provide an integrated and critical assessment of the molecular, experimental, translational and clinical evidence linking aldosterone/MR signaling to atrial remodeling and atrial fibrillation, with particular emphasis on its potential implications in cardiac surgery and postoperative atrial fibrillation. A comprehensive literature search was performed using PubMed/Medline, Scopus and Embase and was updated through September 2026. The exact date of the final literature search was 8 September. No restrictions regarding publication date or language were applied. The search strategy included combinations of terms related to aldosterone and mineralcorticoid receptor signaling (“aldosterone”, “mineralcorticoid receptor”, “primary aldosteronism”, “aldosterone excess”), atrial remodeling and arrhythmogenesis (“atrial fibrillation”, “atrial remodeling”, “atrial cardiomyopathy”, “atrial fibrosis”, “electrical remodeling”, “oxidative stress”, “inflammation”) and cardiac surgery (“cardiac surgery”, “postoperative atrial fibrillation”, “POAF”). To improve reproducibility, a representative search strategy was formulated as follows: ((“aldosterone”[Title/Abstract] OR “mineralocorticoid receptor”[Title/Abstract] OR “primary aldosteronism”[Title/Abstract]) AND (“atrial fibrillation”[Title/Abstract] OR “atrial remodeling”[Title/Abstract] OR “atrial fibrosis”[Title/Abstract] OR “postoperative atrial fibrillation”[Title/Abstract])). Additional targeted searches were performed for specific molecular pathways and emerging translational mechanisms discussed in this review. Original experimental and clinical studies, systematic reviews, meta-analyses and relevant international guidelines and consensus documents were considered. Priority was given to peer-reviewed studies that provided direct mechanistic, translational or clinically relevant evidence concerning the relationship between aldosterone/MR signaling, atrial remodeling, AF and POAF. Landmark studies and recent high-quality publications were preferentially included when multiple sources addressed the same biological or clinical question.

The methodological strength and relevance of clinical studies were evaluated considering study design, sample size, patient selection, appropriateness of comparator groups, adjustment for potential confounding factors and the reliability of outcome assessment. Particular attention was given to whether studies directly assessed endocrine status, atrial remodeling, incident AF or POAF. Randomized controlled trials were considered the most informative for evaluating therapeutic efficacy, whereas observational studies were interpreted primarily as evidence of clinical association. For experimental investigations, particular attention was given to the biological model employed, the specificity of the molecular pathways investigated and whether findings were obtained in human atrial tissue, experimental atrial models or other cardiovascular tissues. These considerations informed the qualitative interpretation of the literature rather than constituting a formal risk-of-bias assessment.

Reference lists of key articles and reviews were also screened to identify additional relevant publications. Titles and abstracts were screened by D.E.T and C.P. and potentially relevant full-text articles were assessed for their contribution to the objectives of the review. Any disagreements regarding study selection or interpretation were resolved through discussion and consensus between the two authors.

Studies were selected according to their relevance to one or more of the following domains: molecular and cellular mechanisms of aldosterone/MR-mediated cardiac or atrial remodeling; experimental evidence linking aldosterone or MR activation to atrial structural or electrical remodeling; clinical associations between primary aldosteronism, renin-independent aldosterone secretion, MR activity and atrial fibrillation; and perioperative evidence linking aldosterone/MR signaling or MR antagonism with atrial remodeling and POAF in patients undergoing cardiac surgery. Studies addressing cardiovascular MR biology without direct atrial relevance were included only when they provided mechanistic information necessary to interpret atrial-specific findings.

Given the narrative nature of the review, no formal meta-analysis or quantitative evidence synthesis was performed. Evidence from experimental models, human observational studies and clinical investigations was critically integrated, with particular attention to the distinction between mechanistic evidence, clinical association and causal inference. Discordant findings were retained and discussed rather than selectively excluding studies that did not support the proposed biological framework. Particular attention was devoted to the cardiac surgical setting. Studies investigating preoperative aldosterone concentrations, MR antagonist exposure, atrial molecular response and POAF were evaluated to determine the extent to which available evidence supports an interaction between a pre-existing aldosterone/MR-mediated atrial substrate and perioperative stress.

For studies specifically addressing cardiac surgery, the interpretation also considered differences in surgical procedures, duration and dosage of perioperative MR antagonist exposure, postoperative rhythm monitoring and potential confounding by indication. Findings from randomized trials, observational cohorts and human atrial tissue experiments were evaluated separately to avoid attributing equivalent evidentiary strength to therapeutic, associative and mechanistic results.

Because the concept of “occult aldosterone excess” is not an established diagnostic entity, evidence concerning subclinical or renin-independent aldosterone secretion was interpreted within the terminology used in the original studies. In this review, “occult aldosterone excess” is therefore used only as a conceptual framework for potentially unrecognized biologically relevant aldosterone/MR activity and not as a formal diagnostic category. Overall, the evidence was synthesized with the aim of distinguishing established biological mechanisms and clinical associations from emerging translational hypotheses, particularly the proposed role of aldosterone-related phenotypes in perioperative atrial vulnerability and susceptibility to POAF.

## 5. Future Directions

Future research should determine whether aldosterone dysregulation represents a measurable and clinically relevant determinant of atrial vulnerability in cardiac surgical patients. Prospective studies should evaluate preoperative aldosterone-renin profiles, accounting for medications and clinical conditions that may influence hormonal measurements and distinguishing overt primary aldosteronism from milder forms of renin-independent aldosterone secretion, in relation to postoperative atrial fibrillation. Endocrine assessment should be integrated with established clinical predictors and, where appropriate, advanced characterization of the atrial substrate through left atrial strain, cardiac magnetic resonance and selected circulating biomarkers. An important objective will be to establish whether aldosterone-related phenotypes provide incremental predictive value beyond conventional POAF risk factors and can identify a biologically distinct subgroup of susceptible patients. Only after such an association has been validated should interventional studies investigate whether treatment of confirmed aldosterone excess or targeted mineralcorticoid receptor modulation can reduce postoperative arrhythmic risk. Future research should therefore progress from phenotype identification to prospective validation and, ultimately, targeted intervention, establishing whether aldosterone represents not only a marker of atrial vulnerability but also a potentially modifiable determinant of POAF.

## 6. Conclusions

Aldosterone has emerged as a biologically relevant mediator of atrial remodeling whose cardiovascular effects extend substantially beyond blood pressure and volume regulation. Experimental studies indicate that persistent mineralcorticoid receptor activation promotes interconnected structural and electrophysiological alterations that favor the development of an arrhythmogenic atrial substrate. Experimental evidence supporting these mechanisms is complemented by clinical observations demonstrating a disproportionate burden of atrial fibrillation and cardiovascular remodeling in patients with primary aldosteronism. These findings support a conceptual framework in which aldosterone/MR-mediated remodeling may contribute to an atrial cardiomyopathic phenotype associated with increased susceptibility to AF. Importantly, milder forms of renin-independent aldosterone secretion may contribute to atrial disease beyond overt primary aldosteronism, whereas tissue MR activation without documented aldosterone excess represents a distinct biological mechanism. Cardiac surgery provides a clinically relevant setting in which this hypothesis may have particular translational relevance. A pre-existing aldosterone-mediated substrate may increase atrial vulnerability to the acute perioperative stress associated with surgery, thereby contributing to the development of POAF. However, this relationship remains biologically plausible rather than clinically established and direct evidence linking preoperative aldosterone dysregulation to POAF is currently limited. Furthermore, conflicting findings from studies investigating perioperative MR antagonism do not currently support its routine initiation solely for POAF prevention. Establishing whether aldosterone-related phenotypes identify patients at increased arrhythmic risk could ultimately provide a mechanistic basis for more individualized strategies of AF prediction and prevention.

## Figures and Tables

**Table 1:** Selected experimental, human atrial, clinical and perioperative evidence. AF: atrial fibrillation; MR: mineralcorticoid receptor; MRA: mineralcorticoid receptor antagonist; PA: primary aldosteronism; POAF: postoperative atrial fibrillation; 11 β-HSD2: 11 β-hydroxysteroid dehydrogenase type 2; CABG: coronary artery bypass grafting; CI: confidence interval; CTGF: connective tissue growth factor; LVEF: left ventricular ejection fraction; NYHA: New York Heart association; HR: hazard ratio; OR: odds ratio; PA: primary aldosteronism; ROCK: Rho-associated protein kinase; snRNA-seq: single-nucleus RNA sequencing.

Study	Design/Population	Main Findings	Relevance to Aldosterone–AF Axis
Hundemer et al., 2018 [7]	Observational PA cohorts: medically treated PA (*n* = 195), surgically treated PA (*n* = 201) and essential hypertension (*n* = 40,092)	Medically treated PA patients with persistently suppressed renin had a higher risk of incident AF than patients with essential hypertension (adjusted HR 2.55, 95% CI 1.75–3.71). No statistically significant excess risk was observed whose renin became unsuppressed (HR 1.03, 95% CI 0.54–2.00) or after adrenalectomy (HR 0.75, 95% CI 0.41–1.36).	Suggests that the adequacy of MR blockade, indirectly assessed through renin response, may influence AF risk in PA. However, the observational design does not establish causality or directly measure atrial MR activity.
Alexandre et al., 2016 [12]	Prospective cohort of 137 patients undergoing elective CABG with preserved LVEF	POAF incidence 24.8%; higher preoperative aldosterone with POAF (183 vs. 143 pmol/L; *p* < 0.01).	Prospective circulating biomarker association; aldosterone autonomy not diagnosed.
Lavall et al., 2014 [45]	Human left atrial samples; experimental cellular models and AF mouse model	Human AF atria showed fibrotic changes and altered 11 β-HSD2 expression. Cellular model experiments implicated CTGF, RhoA/ROCK lysyl oxidase and miR-21.	Direct human atrial association supplemented by experimental mechanistic data
Danesh et al., 2025 [46]	Retrospective cardiac surgery cohort (*n* = 19,042); propensity- score matching (298 MRA users vs. 894 non-users); complementary ex vivo experiments and snRNA-seq of human atrial tissue.	Preoperative MRA use was associated with lower POAF incidence (19.8% vs. 31.8%; *p* < 0.001). Ex vivo, canrenone attenuated MR-target gene expression and stress-associated transcriptional responses in human atrial cells, particularly cardiomyocytes.	Provides complementary clinical and human atrial molecular evidence supporting a potential protective role of MR antagonism. However, residual confounding limits causal inference from the observational cohort, while ex vivo findings do not establish clinical efficacy in preventing POAF.
Reil et al., 2012 [47]	Experimental atrial model with chronic aldosterone exposure (rats receiving chronic aldosterone infusion for 8 weeks, 1.5 mcg/h)	Increased atrial fibroblasts density, interstitial collagen deposition and conduction delay. Atrial arrhythmia inducibility was higher in treated rats (11/11 vs. 2/9 controls; *p* = 0.03) without significant changes in atrial pressure or ventricular function.	Provides direct experimental evidence that chronic aldosterone exposure promotes structural and electrophysiological atrial remodeling independently of detectable hemodynamic alterations in this animal model. Translation to human AF and POAF remains unproven.
Tsai et al., 2021 [84]	Systematic review and pooled analysis; 3 studies; PA *n* = 2705	New-onset AF was more frequent in MRA-treated PA than after adrenalectomy (OR 2.83; CI 1.76–4.57) and more frequent than in essential hypertension (OR 1.91; CI 1.11–3.28)	Comparative observational evidence; treatment selection confounding persists.
Pretorius et al., 2012 [86]	Double-blind placebo-controlled cardiac surgery RCT; *n* = 445	POAF with spironolactone 25 mg/day, ramipril or placebo: 25.9%, 27.8%, 27.2% (*p* = 0.95); therapy begun 4–7 days preoperatively.	Demonstrates that biological plausibility does not automatically translate into efficacy of short-term perioperative MR blockade; important counterbalancing evidence.
Farzaneh et al., 2025 [87]	Single-blind placebo-controlled CABG RCT; *n* = 130	Spironolactone 50 mg/day for 2 weeks: POAF 7.7% vs. placebo 20% (OR 0.33; *p* = 0.042)	Positive perioperative result limited by small trial and single blinding.
Swedberg et al., 2012 [88]	EMPHASIS-HF RCT; NYHA II systolic heart failure; LVEF < 35%; no baseline AF/flutter	Incident AF/flutter 25/911 (2.7%) with eplerenone vs. 40/883 (4.5%) with placebo; HR 0.58 (95% CI 0.35–0.96); *p* = 0.034.	Prespecified secondary arrhythmia outcome; not a cardiac surgery trial.
Shantsila et al., 2020 [89]	IMPRESS-AF double blind RCT; *n* = 250; permanent AF and preserved LVEF	Spironolactone 25 mg/day vs. placebo: no significant change in peak oxygen consumption, E/e’ or quality of life	Not incident AF or POAF prevention; endpoint differs from EMPHASIS-HF

## Data Availability

No new data were created or analyzed in this study. Data sharing is not applicable to this article.

## Abbreviations

The following abbreviations are used in this manuscript:

AF	Atrial fibrillation
POAF	Postoperative atrial fibrillation
MR	Mineralcorticoid receptor
CPB	Cardiopulmonary bypass
MRA	Mineralcorticoid receptor antagonist
